# The Moderating Role of Surgency, Behavioral Inhibition, Negative Emotionality and Effortful Control in the Relationship between Parenting Style and Children’s Reactive and Proactive Aggression

**DOI:** 10.3390/children9010104

**Published:** 2022-01-13

**Authors:** Nora del Puerto-Golzarri, Aitziber Azurmendi, María Rosario Carreras, José Manuel Muñoz, Paloma Braza, Oscar Vegas, Eider Pascual-Sagastizabal

**Affiliations:** 1 Department of Basic Psychological Processes and Their Development, Faculty of Psychology, University of the Basque Country (UPV/EHU), 20018 San Sebastián, Spain; nora.delpuerto@ehu.eus (N.d.P.-G.); o.vegas@ehu.eus (O.V.); eider.pascual@ehu.eus (E.P.-S.); 2 Department of Psychology, University of Cadiz (UCA), 11500 Puerto Real, Spain; rosario.carreras@gm.uca.es (M.R.C.); josemanuel.munoz@gm.uca.es (J.M.M.); paloma.braza@uca.es (P.B.)

**Keywords:** aggressive behavior, temperament, parenting style, children, reactive aggression, proactive aggression, surgency, behavioral inhibition, negative emotionality, effortful control

## Abstract

The principal aim of this study is to explore the moderating role of temperament in the relationship between parenting style and the reactive and proactive aggressive behavior of 8-year-old children. The participants are 279 children (154 boys and 125 girls). To measure reactive and proactive aggression, children completed the reactive and proactive questionnaire (RPQ). Child temperament and parenting styles were evaluated by both parents using the temperament in middle childhood questionnaire (TMCQ) and the parenting styles and dimensions questionnaire (PSDQ). The results revealed that boys with high surgency levels and authoritarian fathers displayed more reactive aggression, whereas behaviorally inhibited boys with mothers who scored low for authoritarian parenting displayed less reactive aggression. Finally, girls with high levels of effortful control and mothers who scored low for authoritative parenting displayed more proactive aggression. The results highlight the value of studying the moderating role of temperament in the relationship between children’s aggressive behavior and both mothers’ and fathers’ parenting styles, and underscores the importance of doing so separately for boys and girls.

## 1. Introduction

Aggressive behavior is viewed as a complex and heterogeneous phenomenon [1] rooted in the interaction of prenatal factors, characteristics associated with the individual characteristics of the child (genetic, physiological and psychological) and various aspects of the social environment [2].

Research focusing on this behavior has identified different subtypes in accordance with different criteria. One of the categories that has been established is based on the function or motive for the aggressive behavior, with a distinction being made between proactive and reactive aggression [3]. Despite correlating closely with each other [4] and being partially overlapped, these two types of aggression differ in terms of their physiological and neurological characteristics [5]. Proactive aggression, also known as predatory or instrumental aggression, is aimed at hurting or harming another person in order to obtain a reward [6] and is characterized by low emotional valence [7]. For its part, reactive aggression, also known as impulsive or spur-of-the-moment aggression, refers to the set of aggressive actions carried out in response to a stimulus that is perceived as threatening or provoking [6]. It is characterized by high emotional valence and the physiological activation of the fight–flight response [7].

Parenting style is a factor that influences children’s aggressive behavior, since their socialization process begins principally with their parents [8]. It is generally accepted that parenting style influences children’s psychological growth, personality development and behavioral adjustment [9,10], having either a positive or a negative effect, depending on the specific style adopted [11]. Children exposed to supportive parenting practices, characterized by warmth, understanding and supervision, have been found to display lower levels of aggressive behavior [12]. Those exposed to hostile parenting styles, on the other hand, characterized by hostile discipline and control, coupled with low levels of warmth and affection, tend to be more aggressive [13,14,15]. These two types of parenting style are categorized in Baumrind’s theory [16] as authoritative and authoritarian, respectively.

The use of hostile and coercive parenting styles has been positively associated with both reactive and proactive aggression [17,18,19], whereas perceptions of positive parental affection have been found to correlate negatively with proactive aggression [20]. Furthermore, in a meta-analysis carried out by Khaleque [21], it was found that there is a negative relationship between perceived maternal and paternal affection and aggressive behavior in children.

Similarly, the use of a parenting style considered to be negative, such as the authoritarian style, has been observed to foster behavioral problems and aggressive conduct [10,22,23,24], whereas children whose parents adopt an authoritative style have higher levels of prosociality and lower levels of aggressive behavior [10,25]. Moreover, several studies have observed sex differences in the effect of parenting style on children’s behavior. Casas et al. [26] found that the authoritative style was associated with lower aggression levels among girls, whereas if mothers adopted a style characterized by psychological control, both boys and girls had higher levels of aggressive behavior. However, if fathers adopted the same style, this only affected aggressive behavior among boys. For their part, Yang, Zhang and Chen [27] found that boys had higher levels of aggressive behavior when exposed to an authoritarian parenting style.

A certain degree of variability has also been observed in the way in which parenting styles affect children, with some authors arguing that this may be due to differences in children’s temperamental characteristics, which act as moderators in this relationship [28,29,30]. Temperament, which has a genetic and neurobiological base [31], is a trait that remains relatively stable throughout a person’s life, although it is susceptible to modification by the environment [32]. This variable has been defined as a set of innate individual differences in reactivity and self-regulation of affect, activity and attention [30]. Based on this definition, different conceptualizations of temperament have been developed, with the most commonly used being that which identifies three dimensions [30,33,34].

The first dimension, negative emotionality, is the tendency to become easily distressed [35] and is characterized by negative emotions, such as fear, anger, distress, irritability, frustration and sadness [36]. Negative emotionality has been found to interact with parenting to predict children’s emotion regulation [37]. When children with high levels of negative emotionality are exposed to a negative parenting style, they have poorer levels of adjustment, which are, in turn, positively associated with externalizing behavior [38,39,40,41]. Furthermore, in a recent study it was found that girls who had a high level of negative emotionality and were exposed to a permissive parenting style exercised by the father showed higher levels of aggressive behavior [42].

The second dimension is surgency, which refers to the predisposition to become actively involved in one’s environment [35]. Surgency includes characteristics such as the absence of shyness, high-intensity pleasure and impulsiveness [43]. Some authors argue that high surgency increases children’s sensitivity to parenting style, prompting them to respond more strongly to the environment [30,44] and develop higher levels of aggression [43,45]. Others, however, suggest that children with low surgency are the ones who are more sensitive to their environment [46], since they may feel overwhelmed by it [47], which could, in turn, be linked to higher levels of aggressive behavior.

The third dimension, effortful control, is understood as an individual’s ability to inhibit their dominant response in favor of sub-dominant ones, thereby enabling them to direct their attention and regulate their emotions and behaviors [30]. As with surgency, effortful control has been associated both positively and negatively with aggressive behavior. Some studies have found that children with low effortful control lack the necessary regulatory tools and so respond to the environment with heightened aggressivity [48]. Others argue that it is children with high effortful control who respond more strongly to socialization influences, since this characteristic enables them to process environmental information more effectively [47], rendering them more sensitive to their surroundings [49].

However, other conceptualizations of temperament have identified a fourth dimension, behavioral inhibition. This is characterized by having a cautious response to new objects, situations or people [50], and by including characteristics such as being shy, quiet, introverted, fearful, and highly cautious [51,52]. Some studies have found that behavioral inhibition inhibits aggression [53], while others have observed that behavioral inhibition is negatively related to direct aggression, trying to avoid confrontation since it can have negative consequences, but positively with displaced aggression since it allows to contain the immediate reactions of the provocation by moving the aggression towards another objective [54,55].

Based on the assumption that the family environment and temperament influence the development of aggression, different theories have been developed within an interactive perspective. Slagt et al. [36] described the theoretical basis for the interactive effects of parenting and temperament in the development of externalizing behavior. In this meta-analysis, they tested whether associations between negative parenting and negative or positive child adjustment (including externalizing problems) as well as between positive parenting and positive or negative child adjustment (including externalizing problems) would be stronger among children higher on putative sensitivity markers. They found that children who had a difficult temperament and children with a high negative emotionality, compared to those children who had an easier temperament and scored low in negative emotionality, were more vulnerable to a negative parenting style. However, these same children benefited more from a positive environment.

One variable believed to predict aggressive behavior that has been studied within different disciplines, including evolutionary biology and developmental psychology, is sex [56,57]. Indeed, many studies have found sex differences in reactive and proactive aggression, with boys scoring higher for both types [17,58,59,60,61]. Some of the different dimensions of temperament that affect behavior have shown sexual differences too, although those differences do not seem to be so clear. Smith and Day [62] observed that high levels of effortful control were associated with lower levels of externalizing behavior among boys, but not among girls.

Sex differences have also been reported in the moderating role played by temperament in the relationship between parenting style and children’s behavior. For example, Carrasco, Delgado and Holgado-Tello [63] found that this relationship was not the same for boys as for girls, and Barnett and Scaramella [64] observed that boys with low fear levels displayed fewer behavioral problems when exposed to a supportive parenting style. For their part, Leve et al. [39] found that harsh discipline predicted higher levels of externalizing behavior only among girls with high impulsiveness and low fear. Nevertheless, other authors have found similar patterns for both sexes [65].

Taking the above into account, we believe that it is important to carry out studies that analyze reactive and proactive aggressive behavior from a biosocial perspective. Thus, we believe that deepening the study of the interactions between temperamental characteristics, such as effortful control and emergence and parenting styles in early stages of development (where there is greater scope for intervention), is very relevant given the contradictory data regarding the direction of such interactions.

Based on these antecedents and the extant research into child aggression in relation to family context and children’s temperament, the present study aims to determine whether possible interactions between temperament dimensions and parenting styles (both maternal and paternal) explain reactive and proactive aggressive behavior among children. Therefore, we predict that:

1. Boys have higher levels of proactive and reactive aggression than girls and we expect to find sex differences also in relation to temperament variables.
2. Children with high or low levels of surgency, who are exposed to a negative parenting (authoritarian or not particularly authoritative parenting style), are more aggressive.
3. Children with high behavioral inhibition, who are exposed to a negative parenting (authoritarian or not particularly authoritative parenting style), are more aggressive.
4. Children with high or low levels of effortful control, who are exposed to negative parenting (authoritarian or not particularly authoritative parenting style), are more aggressive.
5. Children with high negative emotionality, who are exposed to a negative parenting (authoritarian or not particularly authoritative parenting style), are more aggressive.
6. The interactions for predictions 2, 3, 4 and 5 are expected to be different for boys and girls.

## 2. Materials and Methods

### 2.1. Participants

The participants were 279 8-year-old children from the provinces of Gipuzkoa and Cádiz from Spain (154 boys and 125 girls). To recruit the sample, we contacted several schools that were selected, giving priority to the ones with more than one class in the selected year. Once the required consent was obtained from 7 schools (3 semi-private and 4 public), we contacted the parents directly to ask for their permission for their children to participate in the study. Of all the families contacted, 279 signed an informed consent document. According to the knowledge of the area where the participants live, the socioeconomic status of the sample was considered medium and medium-high. All instruments were administered by qualified members of the research team. The project was approved by the Ethics Committee of the institution to which the authors belong, and the procedure complied with the relevant national legislation.

### 2.2. Procedure

At the beginning of the academic year, schools were contacted to request their informed consent. Once the centers accepted their participation in the project, we attended the parents’ meetings that take place at the beginning of the year to inform them about the research and request their participation in it. In addition, a letter was sent to all participating families that included a more extensive explanation of the project, and the informed consent that they had to deliver signed if they wanted to participate. A total of 282 consents were received, of which only 279 were later included in the research due to incomplete data. Through the school, parents were sent a notebook with the paper questionnaires that they had to fill out, namely temperament and parenting styles, so that they could later deliver them back to the school. The aggressive behavior was answered by the children in their own class during school hours.

### 2.3. Aggressive Behavior

Reactive and proactive aggressive behavior was measured using the spanish adaptation of the reactive–proactive aggression questionnaire (RPQ) [66]. Although this questionnaire was originally designed for adolescents, it has successfully been used with 8-year-old children [67,68] as the questions are grammatically simple and these children have the reading ability to understand it. Moreover, every item was read to them aloud. It comprises 23 items divided into 2 dimensions: proactive and reactive aggression. All items are rated on a 3-point Likert-type scale (never, sometimes, often). It is a self-report questionnaire in which subjects rate themselves. The reactive aggression scale comprises 11 items had a reliability of α = 0.74 in our sample. The proactive aggression scale comprises 12 items and had a reliability of α = 0.86 in our sample.

### 2.4. Parenting Style

Parenting style was evaluated using the parenting styles and dimensions questionnaire (PSDQ) [69], which is designed to measure the authoritative, permissive and authoritarian parenting styles. The questionnaire was administered to both mothers and fathers. However, although the parenting style questionnaire was mostly answered by both parents, in some cases the parenting style variable was only obtained for one of them. The instrument comprises 62 items rated on a 4-point Likert-type scale (never, once in a while, very often and always). It was administered to both mothers and fathers in order to assess the frequency with which parents engage in certain behaviors in relation to their child. The authoritative parenting scale, which had a reliability of α = 0.86 in the case of mothers and α = 0.89 in the case of fathers, comprises 27 items. Authoritarian parenting was measured using 20 items and was found to have a reliability of α = 0.76 among mothers and α = 0.70 among fathers. Permissive parenting was measured using 15 items and was found to have a reliability of α = 0.46 for the father and α = 0.60 for the mother in our sample, which was considered very low, so it was not included in the further analyses.

### 2.5. Temperament

Participants’ temperament was assessed using the temperament in middle childhood questionnaire (TMCQ) [70], adapted to the Spanish context by researchers at the University of Murcia [71]. The questionnaire is designed to assess the temperament of children aged between 7 and 10 years, and measures their reactions in a range of different situations over the past 6 months. The questionnaire was completed by both of the children’s parents and comprises 158 items rated on a 5-point Likert-type scale (almost always false, usually false, sometimes true and sometimes false, normally true, almost always true). It provides information about 17 dimensions of temperament, with which a principal components analysis (PCA) was carried out using the varimax rotation (orthogonal). The Kaiser–Meyer–Olkin (K–M–O) measure demonstrated the adequacy of this analysis (K–M–O = 0.756; good value according to Field [72]). Moreover, the Bartlett sphericity x^2^ test ((df = 136) = 1.542112, *p* < 0.001) showed that the correlations between the variables were appropriate for PCA. The analysis identified 4 different factors:

− Factor 1 (Negative emotionality): activation control (−), anger/frustration, attentional focusing (−), discomfort, impulsivity, sadness and soothability/falling reactivity (−).
− Factor 2 (Effortful control): affiliation, fantasy/openness, inhibitory control, low-intensity pleasure and perceptual sensitivity.
− Factor 3 (Surgency): activity level and high-intensity pleasure.
− Factor 4 (Behavioral inhibition): assertiveness/dominance (−), fear and shyness.

Although several previous studies have contemplated only three temperament categories (negative emotionality, effortful control and surgency), in this study we used all four categories identified by the PCA.

### 2.6. Statistical Analyses

First, we checked whether the variables followed a normal distribution and tried to normalize those that did not using the transformation based on Bloom’s ranges [73], which is available as part of the IBM SPSS Statistics for Windows, Version 25.0. Armonk, NY, USA: IBM Corp. However, the reactive and proactive aggression variables could not be normalized using either this or any other method. Consequently, the analyses carried out with these variables were conducted using the bootstrapping technique from IBM SPSS Statistics for Windows, Version 25.0. Armonk, NY, USA: IBM Corp.

Mann–Whitney tests and ANOVAs were performed to check for sex differences among the variables. Next, sex-based Pearson correlations were calculated to explore possible associations between the different variables analyzed in the study. Regression analyses were also carried out to determine the potential moderating role played by temperament in the relationship between parenting style and aggression. All the variables included in the regression models were continuous.

To analyze the interactions that were identified as statistically significant in the regression models, we carried out moderation analyses (model 1) using the PROCESS macro described by Hayes [74] and applying the Johnson–Neyman technique from IBM SPSS Statistics for Windows, Version 25.0. Armonk, NY, USA: IBM Corp.

All statistical analyses were conducted using the IBM SPSS Statistics for Windows, Version 25.0. Armonk, NY, USA: IBM Corp.

## 3. Results

### 3.1. Sex Differences, Means and Standard Deviations

As shown in Table 1, analyses of variance (ANOVAs) and Mann–Whitney U tests were conducted to analyze sex differences in the study variables. The results revealed that boys scored higher for both reactive (U = 7822.000, *p* = 0.003, d = 0.357) and proactive aggression (U= 7649.500, *p* = 0.006, d = 0.445). We also observed a difference in relation to fathers’ authoritarian parenting style, which was more clearly authoritarian when the child in question was a boy (U = 6246.500, *p* = 0.024, d = 0.334).

### 3.2. Correlation Analyses

As shown in Table 2, Pearson correlation analyses were carried out to explore associations between aggressive behavior, temperament factors and parenting styles separately for boys and girls.

### 3.3. Effects of the Interaction between Temperament Factors and Parenting Styles on Aggressive Behavior

To explore whether temperament factors, parenting styles and their interactions had an effect on aggressive behavior, and bearing in mind the sex differences found in the dependent variables, regression models were tested for boys and girls separately, with each model including a temperament factor, maternal or paternal parenting style and the interactions between them. Of the models tested, three were found to be statistically significant and to contain significant two-level interactions. As shown in Table 3, one of the significant models was that which analyzed reactive aggression in boys and contained surgency (R2 = 0.145; F(1,130) = 2.271; *p* = 0.022). In this regression model, a significant two-level interaction was found between surgency and the father’s authoritarian parenting style. The model that analyzed reactive aggression in boys along with behavioral inhibition was also statistically significant (R2 = 0.138; F(1,128) = 2.116; *p* = 0.033), as was the interaction between behavioral inhibition and mother’s authoritarian parenting (Table 4). The last significant regression model analyzed proactive aggression among girls and contained the temperament factor effortful control (R2 = 0.169; F(1,103) = 2.119; *p* = 0.035). In this case, the two-level interaction that was found to be significant was between effortful control and the mother’s authoritative parenting (Table 5).

To analyze significant two-level interactions (Temperament factor * Parenting style), we performed a simple slope test using the PROCESS statistical package from IBM SPSS Statistics for Windows, Version 25.0. Armonk, NY, USA: IBM Corp [74], applying the Johnson–Neyman technique.

**Figure 1:**
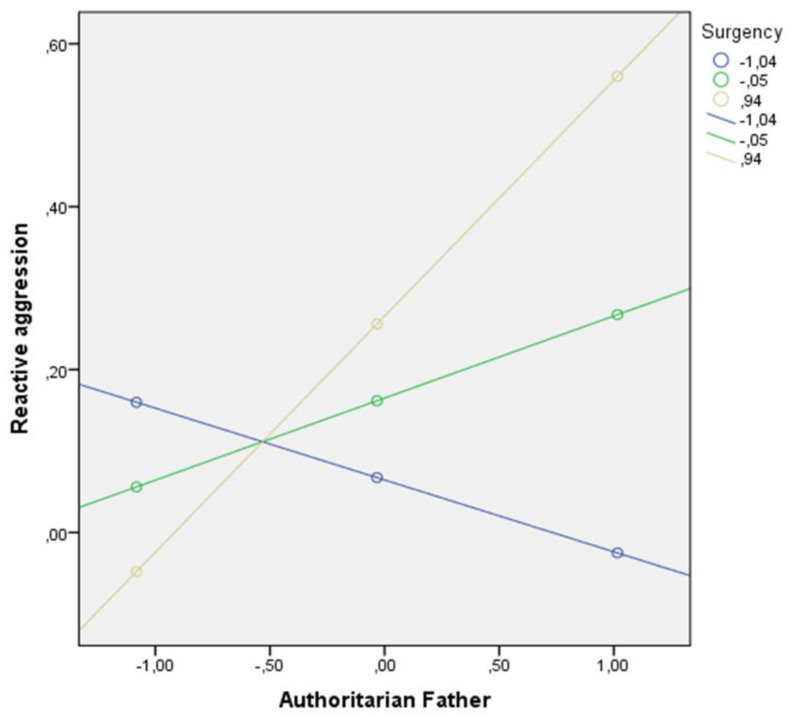
Interaction between surgency and the father’s authoritarian parenting style in relation to reactive aggression in boys.

As shown in Figure 1, the conditional effect of the father’s authoritarian parenting style on boys’ reactive aggression was statistically significant when surgency levels were M ≤ 0.82. In other words, when boys had high levels of surgency, the more authoritarian their father’s parenting style, the more reactive aggression they exhibited.

**Figure 2:**
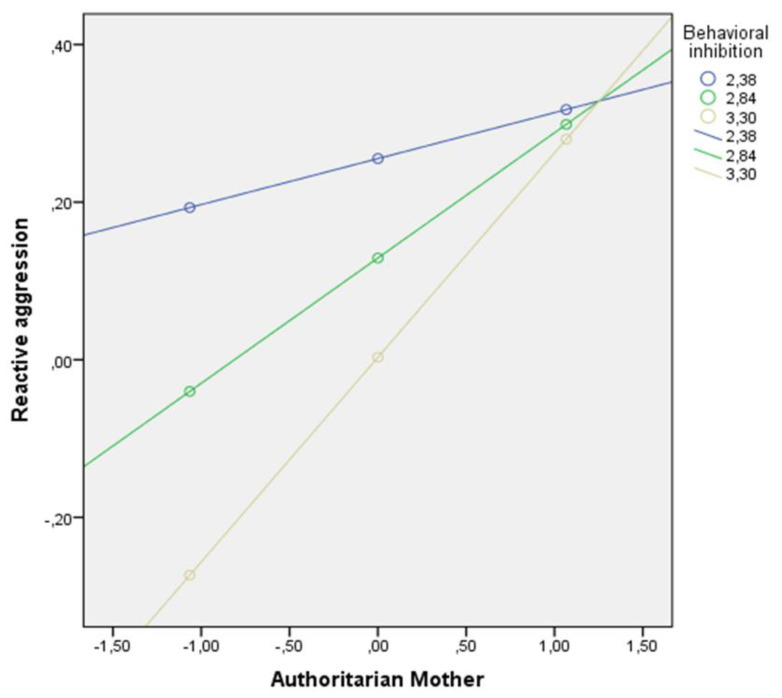
Interaction between behavioral inhibition and the mother’s authoritarian parenting style in relation to reactive aggression among boys.

Upon analyzing the conditional effect of the mother’s authoritarian parenting style on reactive aggression in boys, we observed that it was only statistically significant when behavioral inhibition levels were M ≤ 3.00 (Figure 2). In other words, boys with high levels of behavioral inhibition whose mothers scored low for authoritarian parenting were less reactively aggressive.

**Figure 3:**
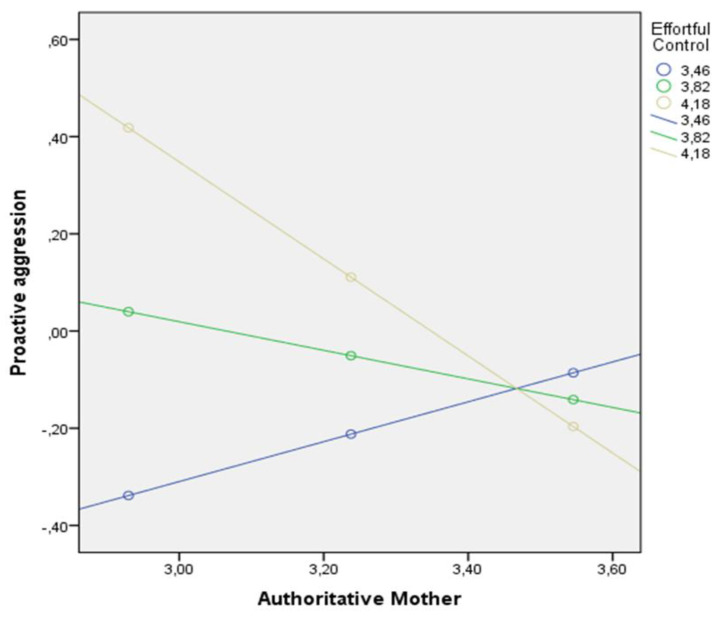
Interaction between effortful control and the mother’s authoritative parenting style in relation to proactive aggression among girls.

Finally, the effect of the mother’s authoritative parenting style on proactive aggression among girls was found to be significant when effortful control levels were M ≤ 3.98. In other words, girls with high levels of effortful control whose mothers scored low for authoritative parenting exhibited more proactive aggression (Figure 3).

## 4. Discussion

The results obtained in the present study indicate that surgency, behavioral inhibition and effortful control moderate the relationship between adverse parenting styles and aggressive behavior among children. Nothing was found with negative emotionality and parenting styles.

Consistent with the results reported by previous studies, our results reveal sex differences in aggressive behavior, with boys having higher levels of both proactive and reactive aggression [17,58,60,61]. There is evidence to indicate that the sex difference observed in relation to reactive aggression is present from birth [75], suggesting that this type of aggression may stem from neurochemical differences between boys and girls. However, as Vitaro et al. [15] point out, it may also be due to the fact that boys’ aggressive behavior is more accepted than girls’, and is even, on occasion, reinforced by the environment. We also observed that fathers were more authoritarian with their sons than with their daughters. This may be due to the possible bidirectional effect between parents’ and children’s behavior. It has been found that children’s behavior influences parenting [76,77], with parents of dysregulated children adopting a more authoritarian style [77] in order to try and force them to act appropriately [78]. It has also been observed that the use of this type of parenting style hampers the development of children’s behavioral control [77]. Consequently, the fact that the boys in our sample were more aggressive than the girls may be related to the difference observed in paternal parenting styles.

As regards the study’s principal aim, upon analyzing the possible interactions between parenting styles and temperament dimensions (negative emotionality, effortful control, surgency and behavioral inhibition), we found two statistically significant interactions that explained reactive aggression in boys and one statistically significant interaction that explained proactive aggression among girls.

Consistent with our second prediction, we observed that boys with high levels of surgency (high activity level and high-intensity pleasure) brought up in what is considered to be a negative family environment (authoritarian father), exhibited more reactive aggression. Several studies have demonstrated that surgency itself, and even the temperament characteristics that make it up, such as high-intensity pleasure, are positively associated with aggressive behavior and the development of externalizing problems [43,45,79]. It has also been widely reported that the authoritarian parenting style is linked to more aggressive behavior [10,24]. However, very few studies have focused on the possible interactive effect between these two variables for explaining aggression, while at the same time considering both the sex of the child and the influence of their mother and father separately. In a recent publication, Wittig and Rodriguez [80] found that infants with high levels of surgency exhibited more externalizing and internalizing problems the more authoritarian their mother’s parenting style was. In our study, it was the father’s authoritarian style that was found to influence aggressive behavior among boys with a temperament characterized by high levels of activity and high-intensity pleasure. In other words, very active boys with high levels of sensation seeking who are brought up by an authoritarian father have high levels of reactive aggression, a type of aggression characterized by being impulsive and unpremeditated.

In relation to the third result found in this study, boys with high levels of behavioral inhibition (low assertiveness and high fear and shyness) brought up by mothers who scored low on the authoritarian parenting scale had low levels of reactive aggression. This finding supports our initial hypothesis, and previous studies have also reported data attesting to the relationship observed. Indeed, several authors have found a negative relationship between the characteristics of this temperament variable (fear, shyness and assertiveness) and externalizing and aggressive behavior [39,81,82,83]. Biederman et al. [82] found that fearlessness in children predicted externalizing behavior, whereas Mofrad and Mehrabi [83] found that high levels of assertiveness led to lower levels of aggressive behavior. Moreover, Acar et al. [81] argued that shyness in children may hamper and inhibit social relationships with peers, since shy children are fearful of participating in social interactions, which in turn leads to lower levels of externalizing behaviors. Gillisen et al. [84] also found that fearful children were more sensitive to parenting style. A maternal parenting style that scores low on the authoritarian scale could be considered more positive than hostile (something which has been negatively associated with aggressive behavior [12]), and may have had a positive effect on the conduct of behaviorally inhibited boys in our sample. Thus, boys who are introverted (and therefore have fewer social relationships) and who have mothers who are not particularly authoritarian were found to exhibit lower levels of reactive aggression.

As regards the fourth of the findings reported here, as hypothesized, girls with high levels of effortful control (affiliation, fantasy, inhibitory control, low-intensity pleasure, perceptual sensitivity) brought up by mothers with low authoritative parenting had higher levels of proactive aggression. This finding is consistent with that reported by Eisenberg, Taylor, Widaman and Spinrad [85], who found a positive relationship between effortful control and externalizing problems. This may be explained by the characteristics of this temperament dimension and the type of aggression in question. The fact that girls with high effortful control have a greater ability to control their emotions and are capable of processing environmental information more effectively may prompt them to engage in aggressive behavior in order to achieve their goals. Moreover, being exposed to a negative parenting style is associated with higher levels of aggressive behavior [13,14,15].

Regarding the fifth of the proposed predictions, no interactive effect was found between negative emotionality and the parenting styles studied when explaining reactive and proactive aggressive behavior. Although it is true that negative emotionality has been related to a poor adjustment based on parenting [36,38,39,40,41], few studies have focused on aggressive behavior. Specifically, a study carried out by Pascual-Sagastizabal et al. [42] found that high negative emotionality moderated the relationship between permissive paternal parenting and girls’ aggressive behavior. However, in this study, the permissive parenting style was not included in the analyses due to its low reliability, which means that the negative context is not fully addressed and therefore could be the cause of not finding significant results.

Finally, the differences observed in temperament dimensions linked to types of aggression in accordance with sex may be explained by the different temperaments that the extant literature associates with each sex. For example, Charbonneau, Mezulis and Hyde [86] reported that girls have higher levels of surgency and better perception and attention skills than boys, which makes them better able to inhibit their impulses and regulate their emotions. For their part, boys prefer high-intensity activities, since their levels of high-intensity pleasure are higher than girls’. Given that we observed no sex differences in the temperament categories used in this study, these assertions should be taken with caution, although they do suggest that interactions between temperament and context may be influenced by sex.

## 5. Conclusions

The results of the present study highlight the importance of taking interactions between children’s temperament and parenting styles into consideration when exploring child aggression. The findings indicate that temperament, which has a biological base, lends variability to the context, and it is therefore vital to study it. Moreover, our results highlight the value of studying these interactions separately for each sex, since, in addition to neurobiological differences, girls and boys may differ also in temperament [49], as well as in terms of how their behavior is affected by the parenting style to which they are exposed [26,27]. The moderating role of individual characteristics in the relationship between context and behavior may be different for each sex. Few studies have focused on this issue to date [62,63], with most using either mixed [49,87] or single-sex samples [88,89].

The present study also emphasizes the importance of studying the parenting styles of both mothers and fathers, since as reported also by other authors [90,91,92], we found that both maternal and paternal parenting styles have an effect on children’s aggressive behavior. Given that it has been observed that parents’ behavior affects children’s temperamental characteristics and behavior [84,93], and since temperament is modulated by context, experience and social interactions [30], we believe that the results reported in this paper may be useful for helping families to develop more appropriate parenting practices as a means of both reducing their children’s aggressive conduct and avoiding the development of other behavioral problems. This is really important as aggressive children are considered to be at increased risk of lifelong disadvantages, as well as this behavior having been considered a public health and social issue. Therefore, prevention of aggression can produce great benefits [2].

Nevertheless, this study has some limitations that should not be overlooked. Firstly, it is important to clarify that the results found cannot be generalized, since the study sample is not representative. Secondly, statistical analysis can be improved by increasing the sample so that the regression models can be reduced and thus reduce the possible experiment wise-error. Finally, these moderation analyses do not allow us to make comparisons by sex and determine whether temperamental characteristics and parenting styles interact differently based on sex when explaining aggressive behavior.

Future research would have to take into account these limitations and also consider that these variables could be analyzed from the susceptibility models proposed by Belsky [94,95].

## Figures and Tables

**Table 1:** Sex differences, means and standard deviations.

	Total (*n* = 279)		Girls (*n* = 125)		Boys (*n* = 154)		F or U	d
	M	SD	M	SD	M	SD		
Reactive aggression	0.680	0.343	0.614	0.315	0.734	0.355	7649.500 **	0.357
Proactive aggression	0.222	0.317	0.148	0.183	0.282	0.384	7822.000 **	0.445
Negative Emotionality	2.747	0.447	2.741	0.434	2.753	0.460	0.026	0.026
Effortful Control	3.788	0.349	3.814	0.356	3.767	0.343	1.229	0.134
Surgency	0.003	1.003	0.047	1.021	−0.031	0.990	0.191	0.078
Behavioral inhibition	2.809	0.492	2.900	0.530	2.848	0.460	0.832	0.104
Authoritative mother	3.212	0.332	3.184	0.322	3.226	0.344	0.498	0.126
Authoritarian mother	1.932	0.310	1.930	0.295	1.942	0.331	7769.500 ^a^	0.038
Authoritative father	3.153	0.372	3.184	0.372	3.130	0.373	1.825	0.144
Authoritarian father	1.899	0.295	1.849	0.279	1.947	0.306	6246.500 *^,a^	0.334

** *p* < 0.01; *p < 0.05; ^a^ Calculated using the Mann–Whitney test.

**Table 2:** Correlations between aggressive behavior, parenting styles and temperament variables (boys above and girls below).

	1.	2.	3.	4.	5.	6.	7.	8.	9.	10.
1. Reactive aggression	-	0.650 **	0.132	0.152	0.133	−0.107	0.083	0.167	0.126	0.037
2. Proactive aggression	0.515 **	-	0.172 *	0.081	0.049	0.064	−0.096	0.066	0.154	0.074
3. Negative Emotionality	−0.100	−0.096	-	−0.123	0.048	0.313 **	−0.126	−0.233 **	0.420 **	0.328 **
4. Effortful Control	0.237 *	0.152	−0.137	-	0.237 **	0.042	0.397 **	−206 *	−0.147	−0.085
5. Surgency	−0.087	−0.021	0.114	0.070	-	−0.305 **	0.170 *	−0.026	0.065	0.041
6. Behavioral inhibition	−0.217 *	−0.017	0.346 **	−0.112	−0.099	-	−0.054	0.004	0.131	0.146
7. Authoritative mother	0.030	−0.029	−0.147	0.474 **	0.253 **	−0.127	-	0.435 **	−0.296 **	−0.204 *
8. Authoritative father	0.211 *	0.109	0.015	0.267 **	0.059	−0.106	0.339 **	-	−0.215 *	−0.393 **
9. Authoritarian mother	−0.203 *	−0.213 *	0.356 **	−0.275 **	0.214 *	0.204 *	−0.162	−0.126	-	0.402 **
10. Authoritarian father	−0.277 *	−0.017	0.192 *	−0.296 **	189 *	0.219 *	−0.202 *	−0.324 **	0.426 **	-

* p < 0.05; ** *p* < 0.01.

**Table 3:** Regression analysis for boys’ reactive aggression including parenting styles and surgency.

	Beta	t	*p*
Authoritative Mother	0.123	0.386	0.689
Authoritative Father	0.720	2.567	0.022 *
Authoritarian Mother	0.126	1.311	0.189
Authoritarian Father	0.123	1.222	0.282
Surgency	−0.679	−0.612	0.515
Sur *Authoritative Mother	0.219	0.901	0.308
Sur * Authoritative Father	0.029	0.101	0.908
Sur *Authoritarian Mother	−0.132	−1.480	0.174
Sur *Authoritarian Father	0.229	2.445	0.027 *

* p < 0.05; Sur = Surgency.

**Table 4:** Regression analysis for boys’ reactive aggression including parenting styles and behavioral inhibition.

	Beta	t	*p*
Authoritative Mother	−2.210	−1.094	0.261
Authoritative Father	3.080	1.548	0.097
Authoritarian Mother	−1.126	−2.061	0.019 *
Authoritarian Father	1.187	1.852	0.050 *
Behavioral inhibition	−0.325	−0.111	0.903
BI *Authoritative Mother	0.854	1.233	0.223
BI *Authoritative Father	−0.841	−1.206	0.191
BI *Authoritarian Mother	0.444	2.347	0.005 **
BI *Authoritarian Father	−0.368	−1.684	0.077

* p < 0.05; ** *p* < 0.01; BI = Behavioral inhibition.

**Table 5:** Regression analysis for girls’ proactive aggression including parenting styles and effortful control.

	Beta	t	*p*
Authoritative Mother	6.561	2.369	0.011 *
Authoritative Father	−0.344	−0.133	0.899
Authoritarian Mother	−0.910	−0.840	0.403
Authoritarian Father	−0.118	−0.108	0.914
Effortful control	5.724	1.939	0.044
EC *Authoritative Mother	−1.782	−2.484	0.009 **
EC *Authoritative Father	0.139	0.210	0.843
EC *Authoritarian Mother	0.186	0.656	0.513
EC *Authoritarian Father	0.060	0.207	0.826

* p < 0.05; ** *p* < 0.01; EC = Effortful control.

## Data Availability

The data presented in this study are available on request from the corresponding author. The data are not publicly available due to the Ethics Committee of the University to which authors belong.
